# Hyperlipidemia and Atherosclerotic Lesion Development in Ldlr-Deficient Mice on a Long-Term High-Fat Diet

**DOI:** 10.1371/journal.pone.0035835

**Published:** 2012-04-25

**Authors:** Yanling Ma, Wenyi Wang, Jie Zhang, Youli Lu, Wenyu Wu, Hong Yan, Yiping Wang

**Affiliations:** 1 State Key Laboratory of Drug Research, Shanghai Institute of Materia Medica, Chinese Academy of Sciences, Shanghai, China; 2 Central Laboratory, Shanghai Xuhui Central Hospital, Shanghai, China; University of Tor Vergata, Italy

## Abstract

**Background:**

Mice deficient in the LDL receptor (*Ldlr* ^−/−^ mice) have been widely used as a model to mimic human atherosclerosis. However, the time-course of atherosclerotic lesion development and distribution of lesions at specific time-points are yet to be established. The current study sought to determine the progression and distribution of lesions in *Ldlr* ^−/−^ mice.

**Methodology/Principal Findings:**

Ldlr-deficient mice fed regular chow or a high-fat (HF) diet for 0.5 to 12 months were analyzed for atherosclerotic lesions with *en face* and cross-sectional imaging. Mice displayed significant individual differences in lesion development when fed a chow diet, whereas those on a HF diet developed lesions in a time-dependent and site-selective manner. Specifically, mice subjected to the HF diet showed slight atherosclerotic lesions distributed exclusively in the aortic roots or innominate artery before 3 months. Lesions extended to the thoracic aorta at 6 months and abdominal aorta at 9 months. Cross-sectional analysis revealed the presence of advanced lesions in the aortic sinus after 3 months in the group on the HF diet and in the innominate artery at 6 to 9 months. The HF diet additionally resulted in increased total cholesterol, LDL, glucose, and HBA1c levels, along with the complication of obesity.

**Conclusions/Significance:**

Ldlr-deficient mice on the HF diet tend to develop site-selective and size-specific atherosclerotic lesions over time. The current study should provide information on diet induction or drug intervention times and facilitate estimation of the appropriate locations of atherosclerotic lesions in *Ldlr* ^−/−^ mice.

## Introduction

For decades, numerous efforts have been made to determine the pathogenesis and optimal treatments for atherosclerosis, which, together with related complications, presents the leading cause of death in industrialized countries. Clinical and epidemiological studies have led to the identification of the genetic, environmental, and inflammatory risk factors of this disease, including dyslipidemia, hypertension, diabetes, obesity, smoking, high-fat diet, and chronic inflammation [1]. Manipulation of lipids levels and inflammatory factors, and combination therapies constitute the main treatment approaches for atherosclerosis at present [2].

Mouse models have been widely employed to analyze the underlying biological mechanisms and optimal drug interventions of atherosclerosis, in particular, *Apoe* ^−/−^ and *Ldlr* ^−/−^mice [3]. Apoe-deficient mice have been extensively investigated with regard to diet, lipoprotein metabolism, and the atherosclerotic lesion progression process [4], [5], resulting in their wide usage as models to mimic human atherosclerosis. Previous studies have demonstrated that *Ldlr* ^−/−^ mice show moderate lipid levels and develop no or only mild atherosclerosis when fed a regular chow diet, in contrast to dramatically elevated cholesterol levels and prominent lesions all over the aorta after 8 months on the Paigen diet [6], supporting their utility as promising models for investigating the pathogenesis of atherosclerosis [7], [8], [9], [10], [11], [12]. While extensive data have been obtained on lesion development in *Apoe* ^−/−^ mice, atherosclerotic lesion progression over time and distribution of lesions at specific time-points in *Ldlr* ^−/−^ mice remain to be defined.

Hemodynamic forces are recognized as crucial factors determining the susceptibility of vascular sites to atherosclerosis [13]. Mouse models develop atherosclerotic lesions reproducibly at susceptible sites associated with low shear or disrupted flow, particularly the curvature and branch points of the aorta [4], [5]. Lesions in the vascular bed go through the following phases: initiation as fatty streaks, progression into intermediate lesions, and development of advanced lesions vulnerable to rupture [2]. Lesions at different or the same vascular sites but with different “lesion age” may show variable responses to different types of modulation. Accordingly, selection of the appropriate times and locations for evaluation of lesions is of critical importance. More recently, researchers have started to use more than one site or time-point to evaluate lesions [14], [15]. Clearly, the specific distribution of lesions on the vascular bed over time needs to be addressed.

In the current study, we quantified atherosclerotic lesion progression in *Ldlr* ^−/−^ mice fed regular chow or high-fat diets over a long-term period. Lesion locations at specific time-points were additionally identified. Based on the current findings, we have expanded the general conception on lesion progression and determined lesion size and location at multiple time-points in *Ldlr* ^−/−^ mice.

## Materials and Methods

### Ethics Statement

This study has been approved and supervised by Institutional Animal Care and Use Committee (IACUC) of Shanghai Institute of Materia Medica, Chinese Academy of Sciences (IACUC Approval Number: SIMM-2010-12-WYP-06). All procedures performed on animals were conducted in strict accordance with the Guide for the Care and Use of Laboratory Animals (Institute of Laboratory Animal Resources, Commission on Life Sciences, National Research Council). All efforts were made to improve animal welfare and minimize suffering.

### Mice and Diets

Male *Ldlr* ^−/−^ mice (C57BL/6 background, Jackson Laboratory, stock number: 002207) were housed in standard cages (6 mice per cage containing toys) in a temperature- controlled (23±2°C) Specific Pathogen-Free room with 12 h dark/12 h light cycles. Eight week-old mice were randomly divided into two groups. One group was fed a regular chow diet, and the other a HF diet (SLACCAS Co., Ltd. China) containing 21% fat (18% added cocoa butter and 3% fat within the basic diet), 0.15% cholesterol, 7% casein, 7% sucrose, and 3% maltodextrin. All mice had free access to water and food, except during a 14 h fast period prior to blood sample collection. Mice were sacrificed at 0, 1, 3, 6, 9, 12 months after HF or chow diet feeding by cervical dislocation.

### Plasma Lipid, Glucose and Blood HBA1c Determination

Blood samples were obtained from the retro-orbital plexus of 14 h-fasted animals anesthetized with isoflurane. Formed elements and plasma were separated by centrifugation (3000 g, 15 min). Plasma total cholesterol, total triglyceride, LDL, HDL and glucose levels were measured enzymatically with commercial kits obtained from Wako Pure Chemical Industries Ltd. and Sichuan Maker Biotechnology Co., Ltd. The blood HBA1c level from formed elements was determined according to instructions of the HBA1c Assay Kit (Sichuan Maker Biotechnology Co., Ltd.).

### Tissue Preparation for *en face* Analysis

After cervical dislocation, mice were perfused with PBS for a few minutes and subsequently fixed via perfusion with fixation solution (4% paraformaldehyde, 5% sucrose and 20 mmol EDTA) for about 15 min. The heart, along with the attached full-length aorta including most branching vessels, was detached carefully and placed in fresh fixation solution. Artery *en face* analysis was performed, as described previously, with slight modifications [6]. The heart and surrounding adventitial fatty tissue were removed under an anatomic microscope. The aorta was opened longitudinally from the aortic root to iliac bifurcation and pinned on a black silica gel plate. The aorta was rinsed in 70% ethanol after 12 h of fixation, stained with 1% Sudan IV in 50% acetone/35% ethanol for about 10 min, and destained in 80% ethanol for 5 min. The stained aorta was stored in fixation solution until images were obtained. Atherosclerotic lesions in full-length aorta, aortic arch, innominate artery (from the branching point to the Y-shaped bifurcation), thoracic aorta, and abdominal aorta were analyzed and quantified relative to the full-length lumen area, using the Image Pro-Plus 6.0 software.

### Tissue Histology and Immunohistochemical Staining

To prepare sections in the aortic sinus and innominate artery for histological staining, heart and aortic arch were processed as described previously [5], [16]. For lipid Oil-Red O staining, tissues were frozen in OCT compound (Tissue-Tek) and sectioned at 8 µm thickness. Sections were stained with Oil-Red O for lipid and counterstained with hematoxylin. For other histological staining, paraffin sections (5 µm) through the aortic sinus were obtained. Modified Movat's pentachrome stain was performed according to an earlier report [17]. Immunohistochemical staining for macrophages and vascular smooth muscle cell Actin (SM α-Actin) was performed as specified in a previous report [18].

### Statistical Analysis

Values are presented as means ± SD, unless otherwise indicated. *P*<0.05 was considered statistically significant with the Student's *t*-test or one-way ANOVA, followed by Dunnett's Multiple Comparison Test.

## Results

### Plasma lipid, glucose and blood HBA1c Levels

**Figure 1:**
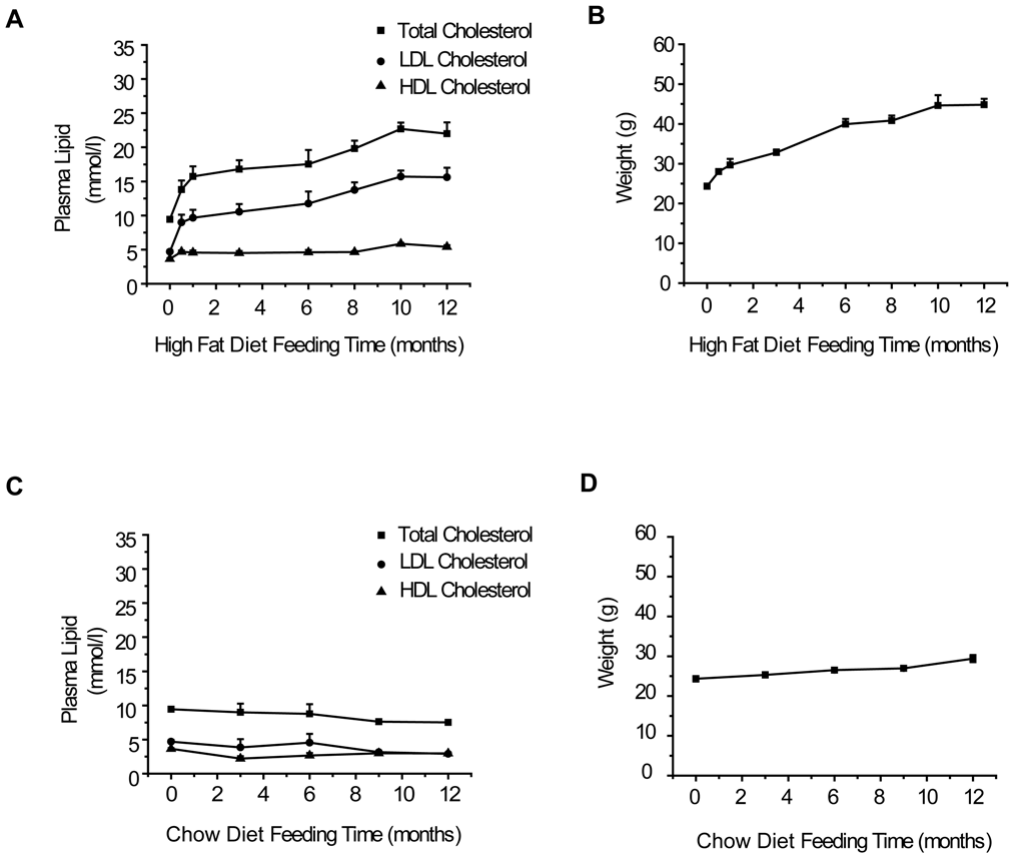
Plasma lipids levels and body weights of *Ldlr* ^−/−^ mice fed HF or regular chow diets over time. Plasma total cholesterol, LDL and HDL levels in HF diet-fed (A), and regular chow diet-fed (C) male *Ldlr* ^−/−^ mice. Body weights of HF diet-fed (B) and regular chow diet-fed (D) mice. Data are presented as mean values ± SEM (n = 10).

As shown in Figure 1A, total cholesterol and LDL levels of *Ldlr* ^−/−^ mice on the HF diet increased dramatically in the first two weeks, followed by a slower increase to final concentrations of 22.00±2.33 mmol/L and 15.60±1.98 mmol/L, respectively. Meanwhile, the HDL level was only moderately elevated with time. Interestingly, the total triglyceride level was not significantly changed over the entire period (Table 1), consistent with previous findings [16], [19]. In contrast, plasma lipids levels were maintained at a steady-state over the 12 months of test time in mice on a regular chow diet (Figure 1C). As reported previously [20], [21], the HF diet induced obesity with an almost one-fold increase in body weight, compared with only 20.8% weight gain in mice fed a regular chow diet (Figures 1B and D) in 12 months.

**Table 1:** Mean plasma TG levels (mmol/L) in *Ldlr* ^−/−^ mice with increasing times of HF or regular chow diet feeding.

Time (months)	HF diet	Regular chow diet
**0**	1.55±0.25	1.55±0.25
**3**	2.00±0.55	1.26±0.23
**6**	1.58±0.69	1.48±1.17
**9**	1.95±0.40	1.47±0.26
**12**	1.80±0.80	1.70±0.49

Since the high-fat diet induced obesity in *Ldlr* ^−/−^ mice, we measured the levels of plasma glucose and blood HBA1c, which serve as the two key markers of diabetes. As shown in Table 2, the HF diet induced a significant increase in glucose and HBA1c levels. Ldlr-deficient mice fed the HF diet for various time-periods displayed similar levels of glucose and HBA1c, which were significantly higher than those of regular chow-fed mice. Comparison of the diet groups revealed HBA1c levels of 3.58%±0.23% and 3.82%±0.19% (t-test, *P*<0.001) and plasma glucose values of 6.92±1.38 mmol/L and 11.56±1.92 mmol/L (t-test, *P*<0.001) for *Ldlr* ^−/−^ mice fed regular chow and the HF diet, respectively.

**Table 2:** Mean HBA1C and glucose levels in *Ldlr* ^−/−^ mice fed a normal or HF diet over time (from 3 to 9 months).

	Time (months)	HBA1C (%)	Glucose (mmol/L)
**Normal diet**	3	3.52±0.21	6.33±1.92
	6	3.57±0.27	7.45±1.16
	9	3.68±0.04	6.99±0.89
**HF diet**	3	3.92±0.12	12.56±2.36
	6	3.76±0.23	10.68±0.61
	9	3.79±0.11	11.05±1.62

### Atherosclerotic lesion progression in full-length aorta

**Figure 2:**
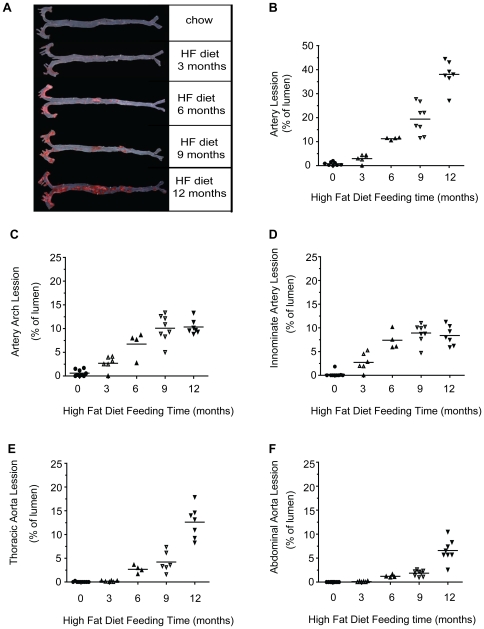
Atherosclerotic lesion progression and location in HF diet-fed *Ldlr* ^−/−^ mice. Analysis of atherosclerotic lesions was performed using the *en face* method. Representative images of Sudan IV-stained aorta in *Ldlr* ^−/−^ mice fed the HF diet for 0 to 12 months (A). Lesion-occupied areas in full-length aorta (A), aortic arch (C), innominate artery (D), thoracic aorta (E), and abdominal aorta (F) were quantified and expressed as a percentage of the lumen area in *Ldlr* ^−/−^ mice on the HF diet at 0 month (•), 3 months (▴), 6 months (▾), 9 months (△), and 12 months (▽).

To investigate the progression of atherosclerotic lesions over time, *Ldlr* ^−/−^ mice fed the high-fat or regular chow diets for various time-periods from 0 to 12 months were sacrificed, and full-length aortas dissected and stained with Sudan IV. As shown in Figure 2, no or extremely slight lesions (<2% of the lumen area) were observed in 0-month HF-fed mice. After 3 months, mice showed slight lesions localized specifically in the aortic sinus, aortic arch or innominate artery. Following relatively slow development over the first 3 months, atherosclerotic lesions were robustly increased in the subsequent months, and extended to the thoracic and abdominal aorta regions at 6 and 9 months, occupying 11.23%±0.52% and 19.40%±6.26% of the lumen areas, respectively. At the end of the study, 38.06%±5.79% of the lumen area was occupied by confluent lesions distributed from aortic sinus to the aortic bifurcation of mice fed the HF diet for 12 months.

Lesions in mice maintained on a regular chow diet were similarly measured over time. Some mice developed slight lesions in the aortic arch as early as 3 months after chow feeding while others did not develop lesions, even after 12 months of being subjected to the same diet (Figure S1). Thus, *Ldlr* ^−/−^ mice fed a chow diet appear to display significant individual differences in lesion development.

### Site selectivity of atherosclerotic lesions over time

To elucidate the relationship between feeding times and lesion location, aortic arch, innominate artery, thoracic aorta, and abdominal aorta were separately evaluated for atherosclerotic lesions. Comparison of locations revealed that lesion initially occurred in the aortic sinus, aortic arch or innominate artery at 0 to 3 months of HF diet feeding. Lesions in these areas increased until they occupied most of the surface at 6 months and maintained a steady-state for the next few months, as shown in Figures 2C and D. Lesions in the innominate artery significantly resemble advanced human atherosclerotic lesions [17]. Detailed morphological and histological changes of lesions in this site are shown in Figure S2. The majority of innominate arteries of *Ldlr* ^−/−^ mice on chow diet were free of lesions with normal morphological and histological characteristics, whereas those of one-month HF diet-fed mice exhibited lipid-loaded macrophages and tunica media thickening. By 9 months, advanced lesions characterized by narrowing vessels, thinning and loss of the fibrous cap, and elastic fragmentation were present (Figure S2).

In contrast to lesions in the aortic arch and innominate artery, those in the thoracic aorta were initially detected in mice after 6 months on the HF diet, and developed further over the next 6 months (Figure 2E). Abdominal aorta lesions subsequent to thoracic aorta lesion development were rarely detected before 9 months of HF feeding, and increased significantly after this time (Figure 2F).

### Atherosclerotic lesion development in the aortic sinus

**Figure 3:**
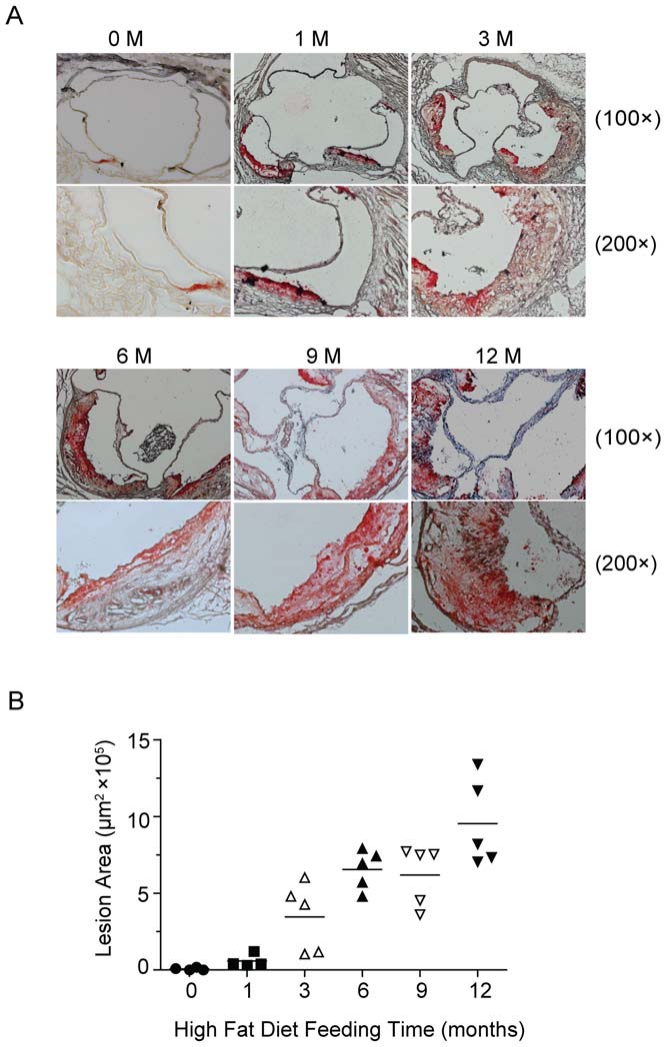
Aortic sinus lesion morphology in *Ldlr* ^−/−^ mice. (A) Representative sections in *Ldlr* ^−/−^ mice fed a regular chow diet (0 M) or HF diet for 1 to 12 months (1 M, 3 M, 6 M, 9 M, and 12 M) stained with Oil Red-O and hematoxylin, original magnification: ×100 or ×200, as indicated; (B) Lesion area was quantified using computer software IPP6.0.

**Figure 4:**
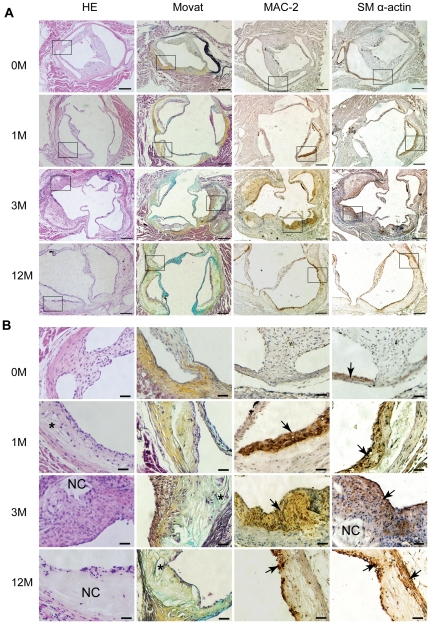
Atherosclerotic lesion development in the aortic sinus of *Ldlr* ^−/−^ mice. A and B, Representative examples of H&E, Movat's pentachrome, VSMC-specific Actin and macrophage antibody (Mac-2)-stained aortic sinus sections are provided. In Movat's pentachrome stained sections, black represents nuclei and elastin fibers, blue represents ground substance and mucin, yellow represents collagen and reticular fibers, red represents muscle, and intense red represents fibrinoid and fibrin. The asterisk indicates the classical cholesterol cleft, while NC stands for necrotic core. The arrow signifies representative regions staining positively for VSMC and macrophage. The bar indicates 200 µm in (A) and 50 µm in (B), respectively.

Cross-sectional analysis provides more information on lesion thickness and its cellular composition. Accordingly, aortic sinuses in *Ldlr* ^−/−^ mice on the HF diet at 0 to 12 months were sectioned and stained with Oil-Red O to detect vascular lipids. Mice on a regular chow diet showed no or small lipid-loaded cells adjacent the valve attachment sites. With increasing HF diet times, lesions progressed to multilayered foam cell deposits, fibrous-cap lesions, or vulnerable thin-cap lesions encircling the aortic sinus (Figures 3A and 4). As shown in Figure 3B, the lesions developed at the greatest rate within 3 months of HF diet feeding. Despite similar lesion thickness, the aortic sinus became ectatic by 6 to 12 months, which is the main cause of the slight increase in lesion size after 3 months on the HF diet. Cross-sectional analysis revealed a parallel lesion development process in the aortic sinus to that in the full-length aorta described previously [22].

As shown in Figure 4, histological examination disclosed a progressive increase in lesion complexity in the aortic sinus. Fatty streak lesions composed predominantly of lipid-loaded macrophages transformed into mature lesions with visible sharp, needle-like cholesterol clefts as early as one month after the HF diet. Numerous lines of evidence support a pivotal role of cholesterol crystals in triggering inflammation [23], [24], [25], and more importantly, occurrence of plague rupture by perforating fibrous tissues [26], [27]. Thus, cholesterol clefts and fibrous cap consisting of VSMCs, extracellular matrix secreted by VSMCs, and small amounts of macrophages (Figure 4) can serve as markers of lesion stability. Thick fibrous-cap lesion with a necrotic core was evident at 3 months. Lesions at this stage were quite stable and less advanced, compared with those observed 6 months or later, characterized by thin fibrous cap, poor collagen, and expanded acellular necrotic core (Figure 4). Notably, lesions in the three individual sinuses (left posterior, right posterior and anterior aortic sinus) may display different development stages, even at the same sample timings (Figure 4, aortic sinus at 1 M and 3 M).

## Discussion

In the present study, we have documented the time-course of hyperlipidemia, hyperglycemia, obesity, and most importantly, atherosclerotic lesions with anatomic location selectivity and diverse sizes, induced by a high-fat diet in *Ldlr* ^−/−^ mice. Thus, based on the development phases and specific locations of lesions, diets or candidate drug intervention times and lesion evaluation sites should be carefully selected.

As reported previously [6], [16], [28], *Ldlr* ^−/−^ mice maintained on a chow diet exhibited no atherosclerosis or only mild lesions with significant individual differences. Fat and cholesterol-enriched diets are commonly used to induce atherosclerotic lesion development. The Paigen diet (1.25% cholesterol, 7.5% cocoa butter, 7.5% casein, and 0.5% sodium cholate) was initially used to induce atherosclerosis in *Ldlr* ^−/−^ mice [6]. However, sodium cholate may cause hepatic steatosis and exerts pleiotropic effects that interfere with the interpretation of data collected during atherogenesis [29]. In a comparison study of dietary cholesterol and cholate content, reproducible and comparable lesions were obtained in *Ldlr* ^−/−^ mice fed semipurified diets supplemented with 0.5% or 1.25% cholesterol or a latter diet containing 0.5% cholate, suggesting that cholate is not necessary for lesion development in *Ldlr* ^−/−^ mice [30]. Teupser and co-workers further determined the role of cholesterol by raising the cholesterol content from 0.00% to 0.5%. The group demonstrated that 0.15% cholesterol is sufficient to induce lesions on the surface of the aorta [16]. Among the established diets [16], [30], [31], [32], [33] to induce atherosclerosis in *Ldlr* ^−/−^ mice, the Western-type diet is the most widely used high-fat regimen for atherosclerosis experiments, usually containing 21% fat and 0.15% to 0.25% cholesterol without cholate [32]. To efficiently induce atherosclerosis and minimize undesirable effects, a high-fat diet containing 21% fat, 0.15% cholesterol and no cholate was employed in the current study.

Compared with *Apoe* ^−/−^ mice, *Ldlr* ^−/−^ mice maintained on either a regular chow or high-fat diet present a moderate model of hypercholesterolemia [3]. An earlier study showed that plasma cholesterol levels were elevated from 5.81±0.70 mmol/L to 40.84±3.10 mmol/L in male *Ldlr* ^−/−^ mice by week 2 of the Paigen diet [6]. In another study, cholesterol attained a new equilibrium level following 4 weeks on the AIN76A semisynthetic diet (0.15% cholesterol) [16]. In the current study, plasma lipids were determined over a longer time-period of high-fat or regular chow diet feeding. Mice showed the most significant increases in plasma lipids levels, including total cholesterol and LDL, in the first month. Contrary to the speculation that plasma lipids reach equilibrium levels within 2 to 4 weeks of a Western-type diet [16], [32], the plasma total cholesterol level kept increasing at a slow rate until 10 months to a final concentration of 22.00±2.33 mmol/L in the current study. Compared with previous data, *Ldlr* ^−/−^ mice developed less severe hyperlipidemia at a relatively slow rate. The different diet components, especially fat and cholesterol, may at least partly account for this discrepancy. However, quantitative differences attributable to the background strains and/or lipid detection assays remain a possibility.

Previous studies on C57BL/6J mice have demonstrated that the aortic sinus is the primary location of atherosclerotic lesion development [34]. Our cross-sectional and *en face* images confirmed this finding in *Ldlr* ^−/−^ mice. Moreover, initiation timing of lesions at different locations were rank-ordered as follows: aortic sinus>innominate artery>thoracic aorta>abdominal aorta. Given that lesions at different sites of the vascular bed have distinct “lesion ages”, they show variable sensitivity to manipulations either attenuating or enhancing atherosclerosis. Inconsistent results were obtained by two different studies focusing on the effects of the pharmaceutical modifier, T-0901317, on atherosclerotic lesion progression [15], [35]. One group used sample timing of 2 months after experimental intervention, while the other selected 3 months. Specifically, in contrast to the significant anti-atherogenic effects on the aortic sinus reported by the first group, the latter study found that T091317 was effective in the innominate artery, rather than the aortic sinus. Based on our data, at the 2 to 3-month time-period, aortic sinus lesions may be too progressive to be affected, while innominate artery lesions, which appear to be initiated later, could be more sensitive to the manipulation. Thus, sampling before lesion progression substantially is necessary to assess the effects of manipulations that influence lesion initiation. In this regard, when using full-length aorta, sample timing should be extended to 3 to 6 months when lesions are in the linear development stage and can be manipulated easily. In contrast, for cross-sectional analysis, we recommended 1 to 3 months as the most susceptible period for lesion initiation study. Furthermore, for lesion regression or stability evaluation, longer diet induction or drug modulation times are required for both *en-face* and cross-sectional analyses. In the present study, lesion with necrotic core and fibrous cap formed by VSMCs and macrophages began to show in one of the 3 individual sinuses at 3 months following initiation of the HF diet (Figure 4), and subsequently transformed into more advanced and vulnerable lesion. Thus, depending on the aim of the experimental manipulation, for example, to prevent initiation of the fatty streak, inhibit lesion progression, or study the regression of advanced plaques in *Ldlr* ^−/−^ mice, it is necessary to induce the appropriate lesion patterns using different time-periods of high-fat diet feeding and careful selection of the anatomic location for lesion analysis.

In summary, *Ldlr* ^−/−^ mice represent a moderate atherosclerosis model that develops lesions in a time-dependent manner with site specificity. In addition, hyperlipidemia, hyperglycemia, and inflammation are induced with a long-term high-fat diet. Our current data obtained with the *Ldlr* ^−/−^ mouse model may provide information on diet induction or drug intervention times and facilitate estimation of the specific locations of atherosclerotic lesions in mice.

## Supporting Information

Figure S1 **Atherosclerotic lesions of normal diet-fed** ***Ldlr*** **^−/−^ mice.** Analysis of atherosclerotic lesions was performed using the *en face* method. Representative images of Sudan IV-stained aorta in *Ldlr* ^−/−^ mice fed the chow diet for 0, 3, 9, and 12 months.
(TIF)

Figure S2 **Atherosclerotic lesion development in the innominate artery of** ***Ldlr*** **^−/−^ mice.** Cryosections were stained with Oil-Red O and paraffin sections with H&E, Movat's pentachrome, VSMC-specific Actin and macrophage antibody. In Movat's pentachrome stained sections, black represents nuclei and elastin fibers, blue represents ground substance and mucin, yellow represents collagen and reticular fibers, red represents muscle, and intense red represents fibrinoid and fibrin. The arrow indicates representative regions staining positively for VSMC and macrophage. The bar indicates 100 µm.
(TIF)
