# Supplementary figures and images for: Hyperlipidemia and Atherosclerotic Lesion Development in Ldlr-Deficient Mice on a Long-Term High-Fat Diet
Source: PLoS One. 2012 Apr 25;7(4):e35835. doi: 10.1371/journal.pone.0035835 (PMC3338468; doi:10.1371/journal.pone.0035835)

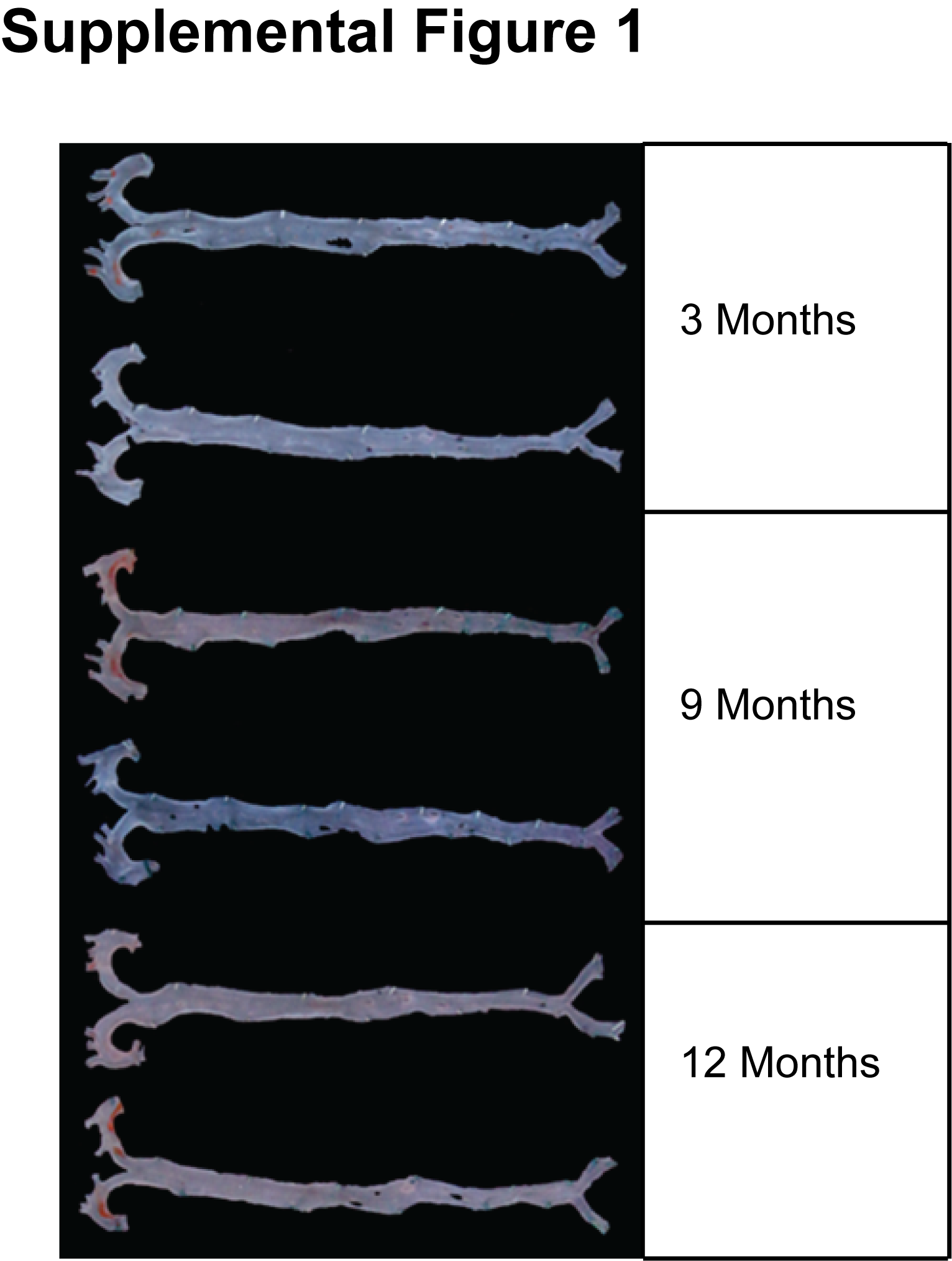

Supplement: Figure S1 — Atherosclerotic lesions of normal diet-fed Ldlr −/− mice. Analysis of atherosclerotic lesions was performed using the en face method. Representative images of Sudan IV-stained aorta in Ldlr −/− mice fed the chow diet for 0, 3, 9, and 12 months. (TIF) [file pone.0035835.s001.tif]

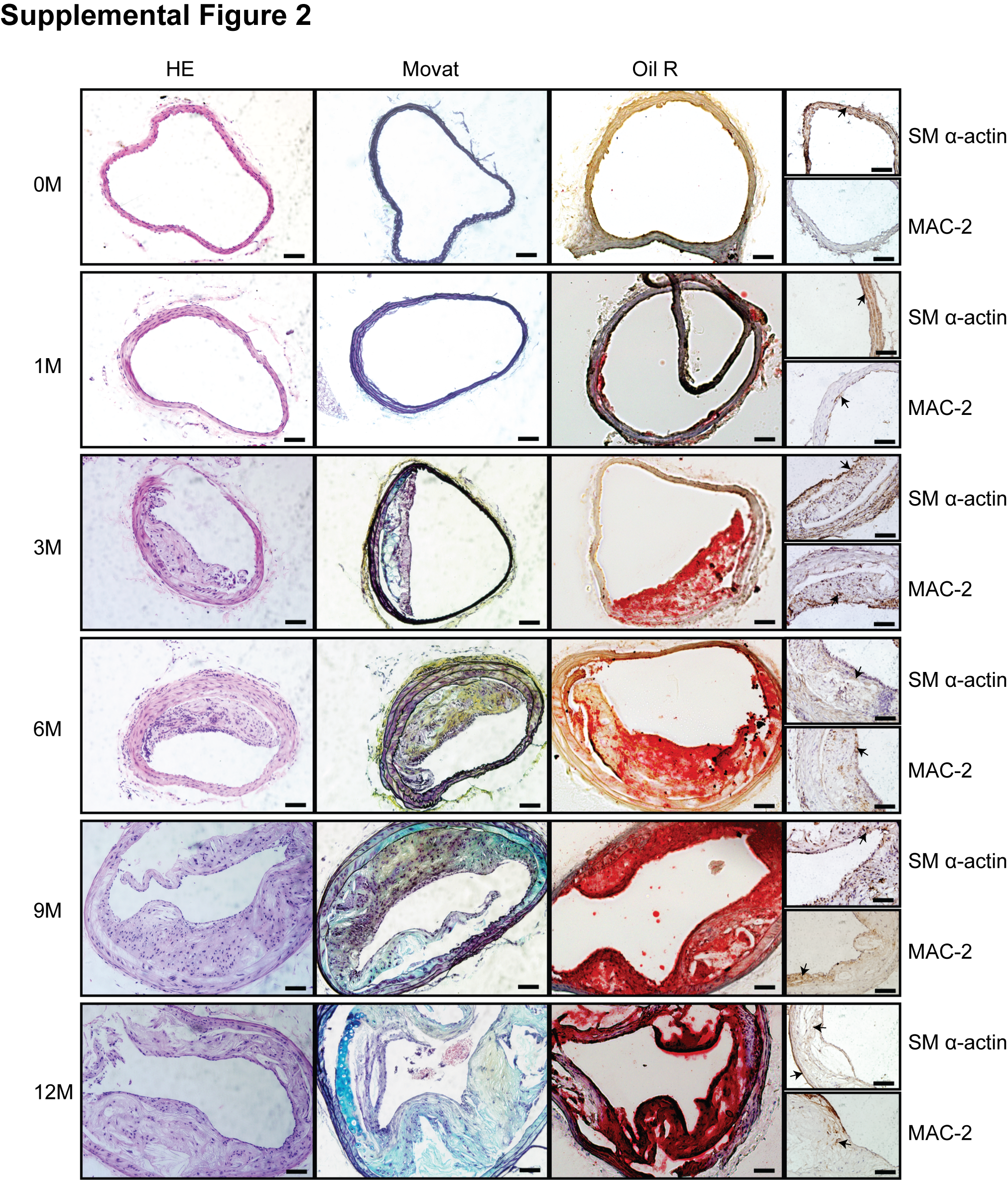

Supplement: Figure S2 — Atherosclerotic lesion development in the innominate artery of Ldlr −/− mice. Cryosections were stained with Oil-Red O and paraffin sections with H&E, Movat's pentachrome, VSMC-specific Actin and macrophage antibody. In Movat's pentachrome stained sections, black represents nuclei and elastin fibers, blue represents ground substance and mucin, yellow represents collagen and reticular fibers, red represents muscle, and intense red represents fibrinoid and fibrin. The arrow indicates representative regions staining positively for VSMC and macrophage. The bar indicates 100 µm. (TIF) [file pone.0035835.s002.tif]
